# Supplementary material for: Charge-neutral fermions and magnetic field-driven instability in insulating YbIr3Si7
Source: Nat Commun. 2022 Jan 19;13:394. doi: 10.1038/s41467-021-27541-9 (PMC8770758; doi:10.1038/s41467-021-27541-9)
Supplement: Supplementary file 1 — Supplementary information. [file 41467_2021_27541_MOESM1_ESM.pdf]

Supplementary Information: Charge-neutral fermions and magnetic field-driven instability in insulating $YbIr_3Si_7$

Y. Sato[1,5], S. Suetsugu[1], T. Tominaga[1], Y. Kasahara[1], S. Kasahara[1,6], T. Kobayashi[1], S. Kitagawa[1], K. Ishida[1], R. Peters[1], T. Shibauchi[2], A. H. Nevidomskyy[3], L. Qian[3,4], E. Morosan[3,4], Y. Matsuda[1]

[1] *Department of Physics, Kyoto University, Kyoto 606-8502 Japan*

[2] *Department of Advanced Materials Science, University of Tokyo, Kashiwa, Chiba 277-8561, Japan*

[3] *Department of Physics and Astronomy, Rice University, Houston, TX 77005 USA*

[4] *Department of Chemistry, Rice University, Houston, TX 77005 USA*

[5] *Present address: RIKEN Center for Emergent Matter Science (CEMS), Wako 351-0198, Japan.*

[6] *Present address: Research Institute for Interdisciplinary Science, Okayama University, Okayama 700-8530, Japan.*

This file includes:

Supplementary Figure 1

Supplementary Figure 2

Supplementary Figure 3

Supplementary Figure 4

Supplementary Figure 5

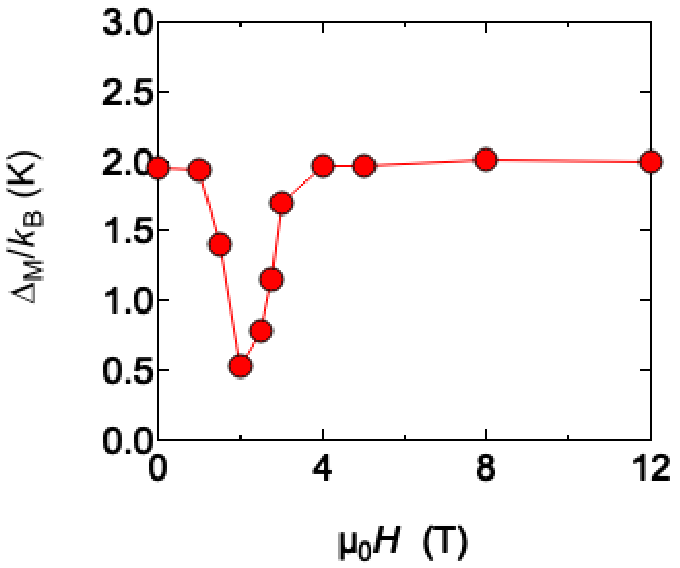

Supplementary Figure 1: **Field dependence of the magnon gap.** The magnon gap Δ_M/k_B is obtained by the fitting of Eq. (1).

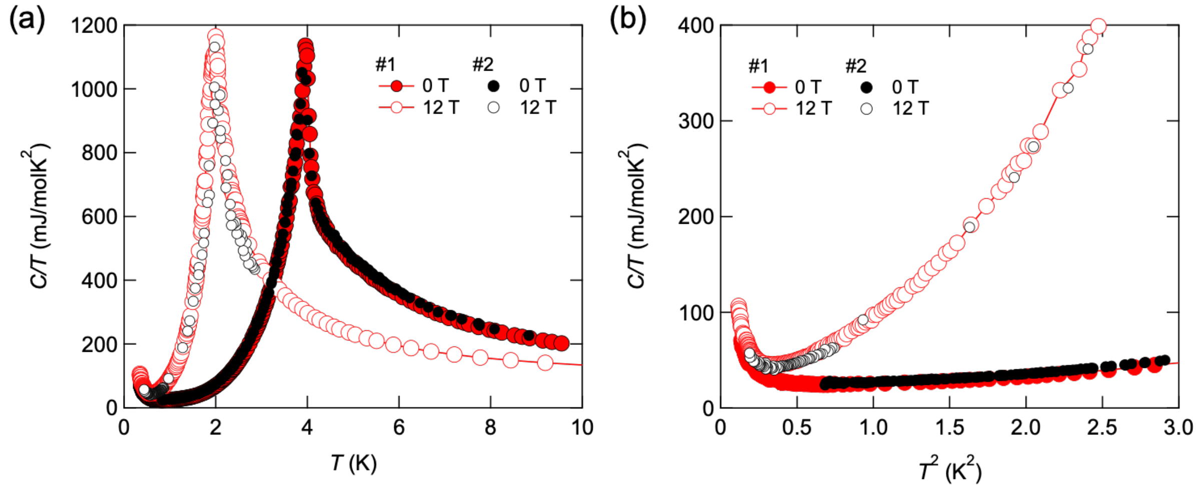

Supplementary Figure 2: **Temperature dependence of specific heat for crystal #2.** (a) Temperature dependence of the specific heat divided by temperature C/T of $YbIr_3Si_7$ crystals #1 and #2 in magnetic field perpendicular to the ab plane. (b) C/T vs. T^2 at low temperatures.

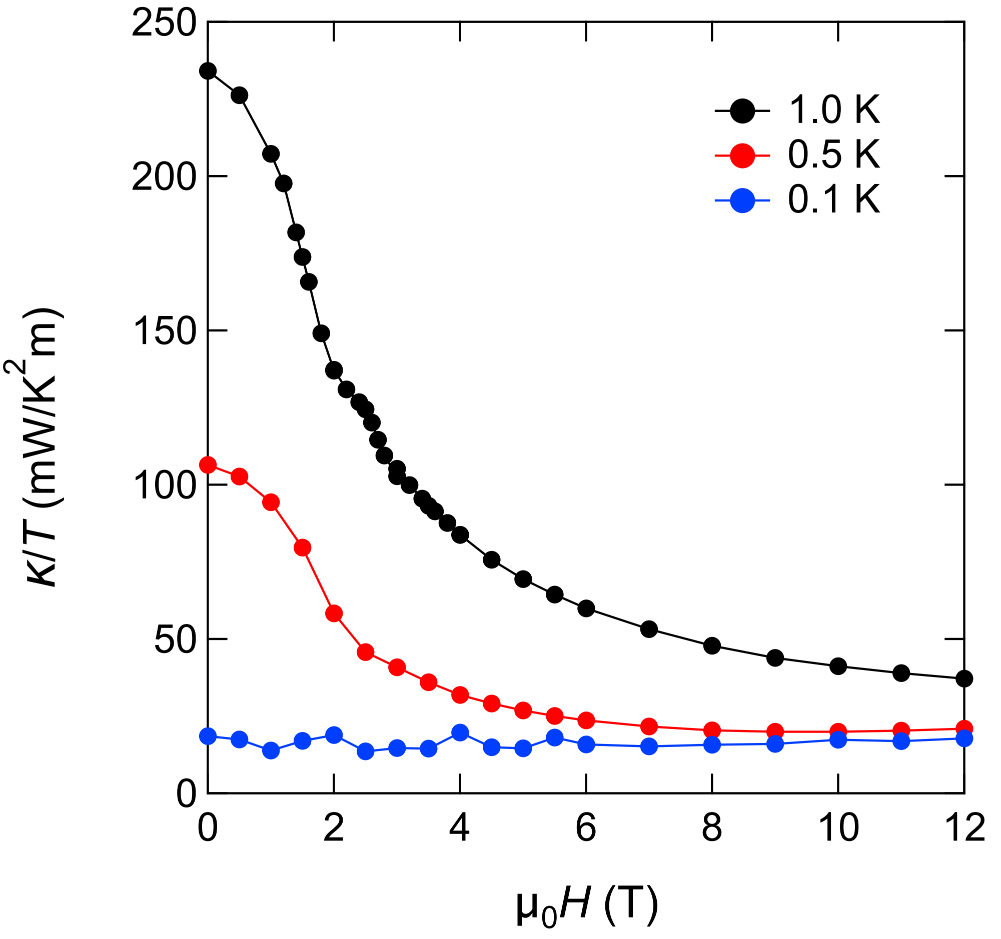

Supplementary Figure 3: **Field dependence of thermal conductivity divided by temperature κ/T.**

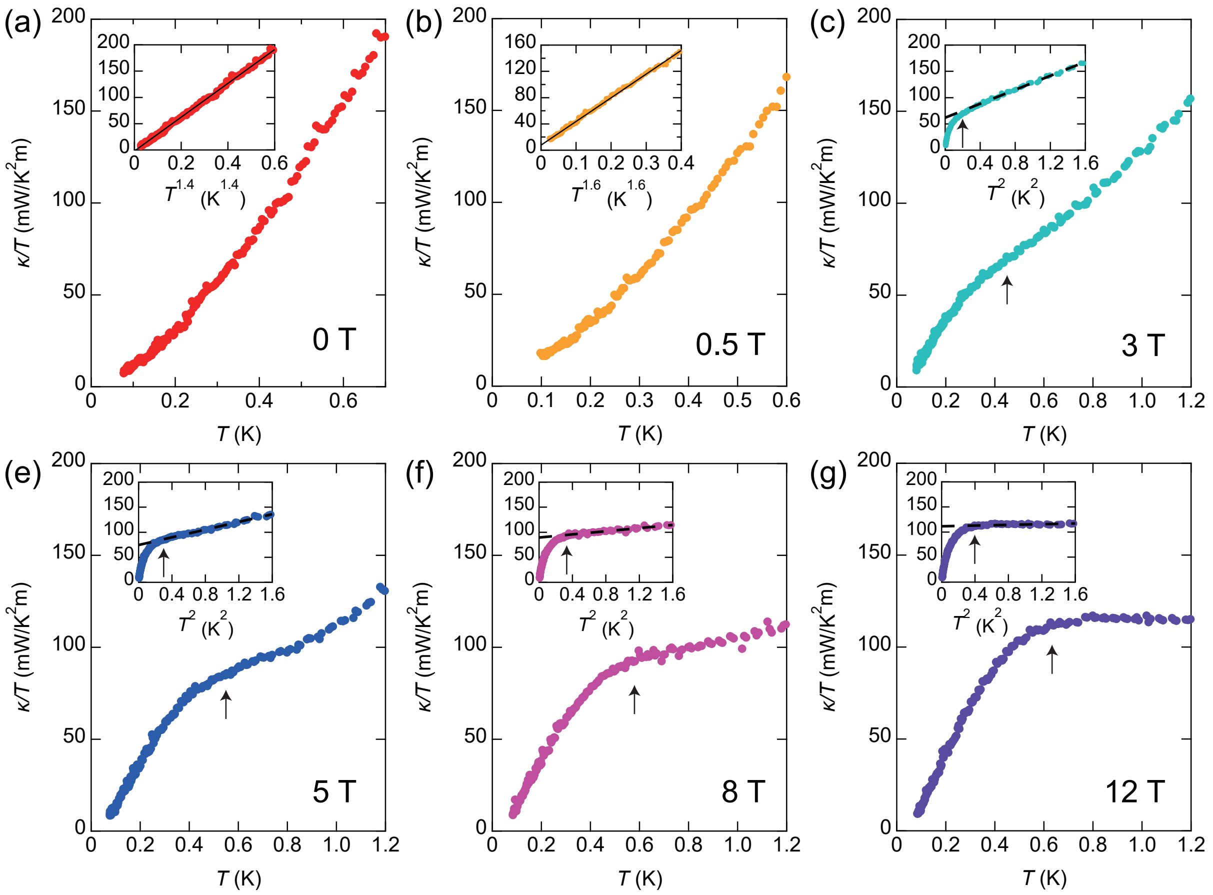

Supplementary Figure 4: **Temperature dependence of thermal conductivity for crystal #2.** Thermal conductivity divided by temperature κ/T of crystal #2 plotted as a function of T in zero and magnetic field for $\boldsymbol{H} \parallel c$ at low temperatures. The insets of (a) and (b) show κ/T plotted as a function of T^p with $p = 1.4$ and 1.6, respectively. The insets of (c)-(g) show κ/T vs. T^2. The solid straight lines of (a) and (b) represent the results of the fitting. The dashed straight lines in (c)-(g) represent the linear extrapolation from the high temperature regimes. Arrows in the main panels and insets indicate the temperatures at which κ/T deviates from the T^2-dependence.

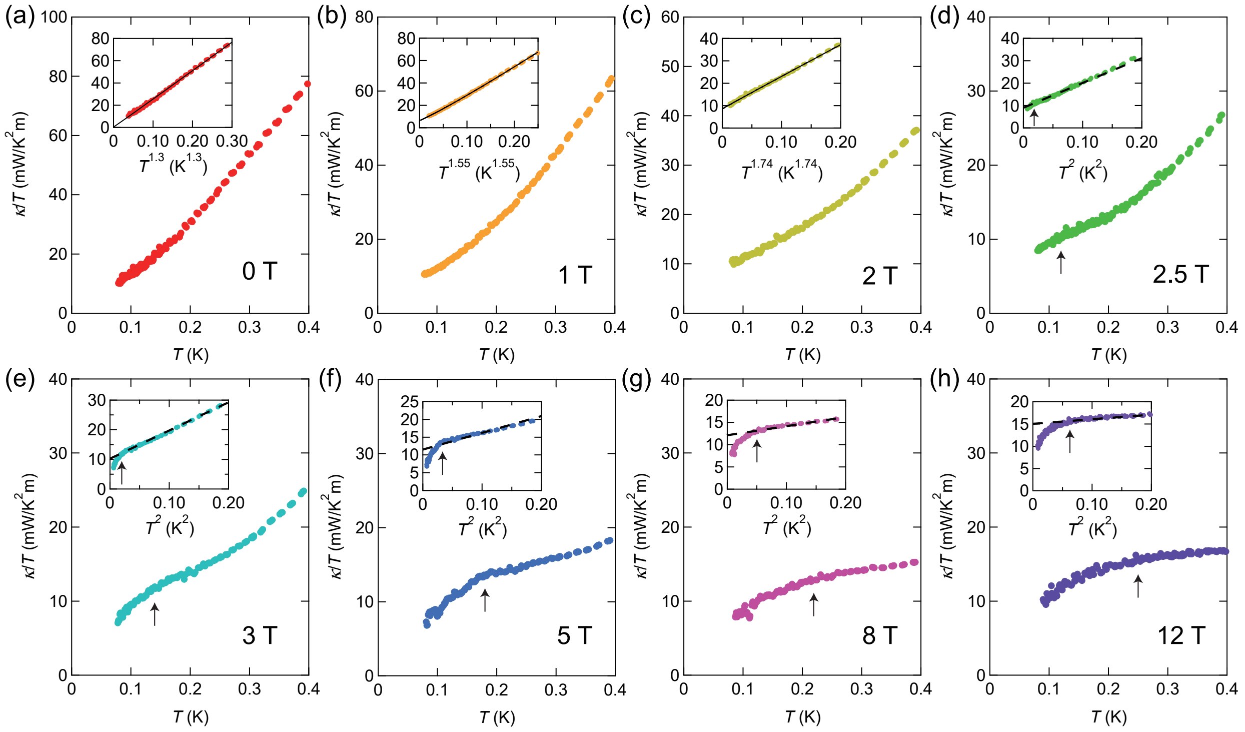

Supplementary Figure 5: **Temperature dependence of thermal conductivity divided by temperature κ/T at low temperature.** (a)-(h) Thermal conductivity divided by temperature κ/T of crystal #1 plotted as a function of T in zero and magnetic field for $\boldsymbol{H} \parallel c$ at very low temperatures. The insets of (a), (b) and (c) show κ/T plotted as a function of T^p with p=1.3, 1.55 and 1.74, respectively. The insets of (d)-(h) show κ/T vs. T^2. The solid straight lines of (a)-(c) represent the results of the fitting. The dashed straight lines in (d)-(h) represent the linear extrapolation from the high temperature regimes. Arrows in the main panels and insets indicate the temperatures at which κ/T deviates from the T^2-dependence.
